# Gender disparities in midlife hypertension: a review of the evidence on the Arab region

**DOI:** 10.1186/s40695-017-0020-z

**Published:** 2017-05-04

**Authors:** Christelle Akl, Chaza Akik, Hala Ghattas, Carla Makhlouf Obermeyer

**Affiliations:** Center for Research on Population and Health, Faculty of Health Sciences, American University of Beirut, P.O. Box: 11-0236, Riad El Solh, Beirut 1107-2020 Lebanon

**Keywords:** Midlife, Women, Gender disparities, Sex ratio, Hypertension, High Blood Pressure, Arab World

## Abstract

**Objective:**

While gender differences in hypertension and increased prevalence rates among women at midlife have been documented in multiple settings, the evidence on the Arab world has not been systematically examined. This review summarizes the evidence related to gender disparities in midlife hypertension in this region.

**Methods:**

We searched MEDLINE and Social Sciences Citation Index (SSCI) databases for studies, published between January 2000 and August 2015, on hypertension in the 22 countries of the Arab region. We abstracted information on the prevalence of hypertension among women and men, in general populations during midlife.

**Results:**

Nineteen studies provided data on the prevalence of hypertension by gender and age in the Arab world. Higher rates of hypertension were found among Arab women at midlife in most countries. In studies that included subjects younger than 35 years old, a decrease in sex ratios (M/F) at midlife was observed in all countries except Palestine. Higher female prevalence rates are observed in the 4^th^ decade of life in most countries of the region, almost two decades earlier than in other parts of the world.

**Conclusions:**

This review highlights the need for more systematic examinations of hypertension in the Arab region, its risk factors, and the reasons for the particular patterns of gender differences that are observed. Such research would have considerable implications for prevention, treatment, and improved well-being.

## Background

High blood pressure (HBP) is the leading risk factor for global disease burden, being responsible for almost 55% of cardiovascular deaths and 8.6% of disability-adjusted life years (DALYs) in 2015 [1–5]. Hypertension is often underestimated among women due to the common belief that cardiovascular disease is a “male” problem [6] — a misperception that still prevails, despite hypertension being the highest risk factor for global mortality among women, accounting for 20.4% of total deaths in 2015 [4]. Overall prevalence of hypertension is similar among women and men, but gender disparities in HBP have been shown to be age-dependent, with a remarkable increase at midlife in rates of hypertension among women as compared to men [7–11]. The National Health and Nutrition Examination Survey (NHANES) 2007–2012 showed that hypertension was higher among males until age 54, similar among males and females from 55 to 64 years of age, and higher among females from age 65 [12]. The mechanisms underlying these age-dependent disparities are not fully understood. Several factors have been invoked, including sex hormones, the renin–angiotensin system, oxidative stress, weight gain and sympathetic activation [6, 13, 14]. While the hormonal alterations observed in women during menopause have been the prevailing hypothesis, epidemiological studies have not been conclusive in this regard, with some studies suggesting that the rise in blood pressure after menopause is age-related, and others arguing that ovarian hormones contribute to an increased risk of hypertension, independently of age [9, 10, 15–18].

Hypertension is particularly high among adults in the Eastern Mediterranean region [1] and is the first risk factor for mortality and morbidity in the North Africa and Middle East region, accounting for 8% of DALYs in 2013, with a remarkable increase of 83% since 1990 [19]. The prevalence of hypertension among Arab middle-aged populations has not been analyzed by gender. This study aims at (1) summarizing available data on the prevalence of hypertension by gender and age, with special attention to the midlife in the Arab world; (2) examining gender disparities related to midlife hypertension in the Arab world; and (3) discussing key factors that could explain these gender disparities.

## Methods

We searched MEDLINE and Social Sciences Citation Index (SSCI) databases for studies published between January 2000 and August 2015, on hypertension in countries of the Arab region. The selection of studies was done as a two-step process. First, studies were eligible for inclusion if they: (1) were conducted among residents of Arab countries (defined as the 22 countries of the Arab League); (2) reported on the prevalence of hypertension, and/or awareness, treatment or control of hypertension; (3) described the study design and methods; and (4) described how hypertension was assessed. Studies published in any language were included. Multi-country studies were included if they presented data on at least one Arab country. Studies conducted exclusively on clinical populations or on individuals suffering from particular diseases, and studies conducted on Arabs residing outside the Arab region were excluded. Additional quality considerations were taken into account: studies that did not report on sample size, age range of study population, methods to assess hypertension, cut-offs values, and studies that presented inconsistent numbers were excluded.

A further selection process was performed to retain only those studies reporting the prevalence of hypertension among general populations by gender during midlife. Midlife is typically defined starting at age 40 and extending to age 60, and studies differ as to how they define the exact beginning and end of midlife [20]. In this analysis, we used slightly wider cut-off points of 35 and 65, reflecting the age ranges of many of the surveys, in order to include the largest number of subjects in the analysis.

For each study, we calculated the sex ratios and present the significance of differences in the sex ratio; p-values are presented either as reported in the original publications, or as calculated based on chi-square tests. All statistical analyses were carried out using STATA version 13 (STATA Corporation, College Station, Texas, USA).

## Results

**Fig. 1:**
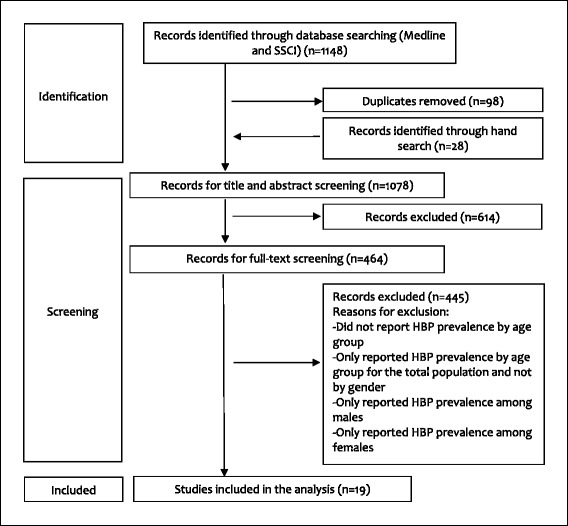
Study flow diagram

Out of 251 articles, 19 fulfilled the eligibility criteria (Fig. 1). They covered 11 out of the 22 countries that are members of the Arab league: Algeria [21, 22], Egypt [23], Jordan[24, 25], Kingdom of Saudi Arabia (KSA) [26, 27], Kuwait [28], Lebanon [29, 30], Oman [31], Palestine [32], Syria [33], Tunisia [34–38], and UAE (United Arab Emirates) [39]. The 19 articles were based on surveys assessing the prevalence of chronic conditions, including HBP among general populations. Five of the studies were based on nationally representative samples; these were conducted in KSA [27], Lebanon [30], Oman [31], and Tunisia [34, 36]. Hypertension was assessed by actual blood pressure measurements in three studies [26, 27, 39]; by either blood pressure measurements or previous diagnosis as reported by respondents in 12 studies[21, 22, 24, 29, 31–38]; and by self-report of HBP in four studies [23, 25, 28, 30].

Table 1 shows the prevalence of hypertension in the Arab region by gender for 16 studies that provided the percent prevalence by gender and age. We present prevalence according to the specific age categories included in the studies, and prevalence during midlife (by selecting age groups from 35 to 65 years wherever possible); the total study sample size is also presented. For all but three studies that had missing data [25, 31, 35], the p-value for the gender difference in prevalence of hypertension is presented. The prevalence of hypertension during midlife could not be pooled across the different studies, due to the disparity in age group categorization and in ways of assessing hypertension.

**Table 1:** Hypertension prevalence by gender and age group in the Arab World (> = 18 years), 2000-2015

Country	First Author (Year of publication)	Sample size ^a^	Age group (years) ^b^	Prevalence of Hypertension (%)											
				By age group				Midlife ^c^				Total sample			
				Male	Female	Ratio M/F	95% CI	Male	Female	Ratio M/F	95% CI	Male	Female	Ratio M/F	95% CI
Self-reported hypertension															
Egypt	Abolfotouh, M.A. (2008) [23]	1800	18–24	0.7	2.1	0.33	[0.04-2.59]	16.6	31.6	0.53***	[0.36-0.78]	7.3	10.5	0.70 *	[0.51-0.94]
			25–44	4.2	7.6	0.55 *	[0.32-0.97]								
			**45–64**	16.6	31.6	0.53***	[0.36-0.78]								
			> = 65	20.0	43.8	0.46	[0.19-1.09]								
Jordan	Kulwicki, A. D. (2001) [25]	209	17-34	2.3	1.6	1.44 ^e^	^e^	11.1	11.4	0.97 ^e^	^e^	7.1	9.6	0.74	[0.29-1.91]
			**35-54**	11.1	11.4	0.97 ^e^									
			55-93	14.3	35.3	0.41 ^e^									
Kuwait	Shah, N.M. (2010) [28]	2487	50-59	30.7	32.0	0.96	[0.77-1.20]	30.7	32.0	0.96	[0.77-1.20]	52.3	49.5	1.06	[0.98-1.14]
			60-69	50.8	55.1	0.92	[0.81-1.05]								
			> = 70	67.0	69.3	0.97	[0.88-1.06]								
Lebanon	Tohme, R. A. (2005) [30]	2010^a^	**30-40**	5.7	5.8	0.98	[0.52-1.86]	22.0	23.1	0.95	[0.75-1.21]	22.1	24.4	0.91	[0.78-1.09]
			**41-50**	13.7	15.5	0.88	[0.58-1.34]								
			**51-60**	31.3	32.6	0.96	[0.73-1.26]								
			61-70	41.0	60.6	0.68 **	[0.53-0.87]								
			>70	53.4	64.6	0.83	[0.63-1.09]								
Diagnosed hypertension based on BP measurements ^d^															
Algeria	Hamida, F. (2013) [21]	722	**40-49**	21.1	20.6	1.02	[0.59-1.77]	29.2	33.3	0.88	[0.63-1.21]	51.3	49.7	1.03	[0.90-1.21]
			**50-59**	40.8	48.1	0.85	[0.58-1.24]								
			60-69	71.4	67.6	1.06	[0.85-1.32]								
			> = 70	75.7	80.6	0.94	[0.80-1.11]								
Jordan	Khader, Y. (2007) [24]	1121	25-44	33.0	32.2	1.02	[0.79-1.32]	^g^				49.5	52.0	0.95	[0.84-1.08]
			45-85	63.7	73.2	0.87 *	[0.77-0.98]								
KSA	Al-Daghri, N.M. (2011) [26]	9149	18 - 45	16.8	12.2	1.38 ***	[1.18-1.61]	41.6	39.9	1.04	[0.93-1.17]	31.5	23.8	1.32 ***	[1.21-1.42]
			**46 - 60**	41.6	39.9	1.04	[0.93-1.17]								
			61 - 80	57.2	61.1	0.94	[0.84-1.04]								
	Al-Nozha, M. M. (2007) [27]	17230^a^	30-39	14.1	10.4	1.36 ***	[1.18-1.57]	28.6	29.6	0.97	[0.90-1.03]	28.6	23.9	1.20 ***	[ 1.14-1.26]
			**40-49**	23.0	24.1	0.95	[0.86-1.06]								
			**50-59**	34.9	39.5	0.88 **	[0.81-0.96]								
			60-70	44.6	50.4	0.88 **	[0.82-0.96]								
Lebanon	Matar, D. (2015) [29]	1697	21-34	20.0	9.0	2.22***	[1.43-3.39]	49.9	37.4	1.33 ***	[1.14-1.56]	42.7	29.5	1.45**^f^	[1.28-1.68]
			**35-49**	42.0	24.0	1.75 ***	[1.33-2.28]								
			**50-64**	60.0	54.0	1.11	[0.94-1.32]								
			> = 65	76.0	68.0	1.12	[0.92-1.37]								
Oman	Al-Riyami A. (2002) [31]	6414^a^	20-39	23.9	16.8	1.42 ^e^	^e^	46.5	50.7	0.92	^e^	35.3	31.3	1.13***^f^	[1.06-1.22]
			40-59	46.5	50.7	0.92 ^e^									
			> = 60	60.9	68.1	0.89 ^e^									
Palestine	Khdour, M. R. (2013) [32]	2077	25-44	17.0	16.7	1.02	[0.75-1.38]	34.3	27.6	1.24 *	[1.03-1.51]	29.2	26.4	1.11**	[0.96-1.27]
			**45-64**	34.3	27.6	1.24 *	[1.03-1.51]								
			> = 65	52.3	48.3	1.08	[0.86-1.37]								
Tunisia	Ben Romdhane, H. (2012) [34]	8007^a^	**35-44**	16.0	14.5	1.10	[0.94-1.30]	24.3	29.4	0.83***	[0.76-0.89]	27.3	33.1	0.82^f^	[0.77-0.88]
			**45-54**	26.3	33.6	0.78***	[0.69-0.89]								
			**55-64**	40.3	53.5	0.75***	[0.67-0.85]								
			65-74	51.1	66.0	0.77***	[0.69-0.87]								
	Ben Romdhane, H. (2005) [35]	1837	**40-49**	21.4	30.4	0.70 ^e^	^e^	^g^				38.7	48.2	0.80***	[0.72-0.90]
			**50-59**	40.5	54.4	0.74 ^e^									
			60-69	56.0	73.0	0.77 ^e^									
	Bouguerra, R. (2006) [36]	3857^a^	20-29	31.3	22.2	1.41 **	[1.10-1.82]	41.3	52.6	0.79***	[0.69-0.89]	45.2	44.0	1.03	[0.96-1.11]
			30-39	28.9	29.5	0.98	[0.78-1.22]								
			**40-49**	34.4	45.5	0.76 **	[0.62-0.91]								
			**50-59**	52.4	63.1	0.83 *	[0.71-0.98]								
			60-69	65.6	75.3	0.87 *	[0.78-0.97]								
			> = 70	76.1	82.5	0.92	[0.82-1.03]								
	Elasmi, M. (2009) [37]	2483	**35-44**	9.0	17.0	0.53***	[0.39-0.75]	22.9	33.0	0.69***	[0.61-0.79]	25.0	36.0	0.69***	[0.62-0.78]
			**45-54**	30.0	43.0	0.70***	[0.59-0.84]								
			**55-64**	37.0	63.0	0.59***	[0.49-0.71]								
			65-69	51.0	78.0	0.65***	[0.51-0.81]								
UAE	Baynouna, L. M. (2008) [39]	817	20-29	5.1	1.7	3.00	[0.32-28.01]	26.2	29.4	0.89	[0.65-1.22]	21.8	19.8	1.10	[0.84-1.44]
			**30-39**	11.1	7.0	1.59	[0.65-4.00]								
			**40-49**	17.0	23.0	0.74	[0.42-1.30]								
			**50-59**	33.6	38.8	0.87	[0.59-1.26]								
			> = 60	36.8	36.7	1.00	[0.57-1.76]								

*BP* blood pressure, *CI* Confidence interval, *F* Female, *M* Male, *SBP* systolic blood pressure, *DBP* diastolic blood pressure

*p <0.05 ** *p* ≤ 0.01; *** *p* ≤ 0.001: significant difference between genders. For the age groups and the midlife age categories, *p*-values were calculated based on chi-square tests assessing the significance of differences in sample proportion. For the total sample, p-values were presented either as reported in the original publications [21, 24, 26–32, 34–37], or calculated based on chi-square tests [23, 25, 39]

^a^Nationally representative studies

^b^The age groups that were selected for each study in order to generate midlife prevalence of hypertension are highlighted in bold.

^c^Prevalence of midlife hypertension by gender was compiled by selecting the age groups falling between 35 to 65 years in the original publications

^d^Hypertension was assessed by actual BP measurements [26, 27, 39], or by either BP measurements or self-report of previous diagnosis [21, 24, 29, 31, 32, 34–37]. For BP measurements, hypertension was identified when SBP > =140 and/or DBP > =90 mmHg for all studies except for Khader et al., 2007 [24] and Bouguerra et al., 2006 [36], where the cut-offs used were SBP > =130 and/or DBP > =85 mmHg

^e^Significance between genders and 95% confidence intervals could not be calculated due to insufficient data [25, 31, 35]

^f^The significant difference between genders for the total sample, reported from the original publications, was adjusted in three studies [29, 31, 34]

^g^Prevalence of midlife hypertension could not be determined due to overlapping age ranges [24], or insufficient data to generate pooled prevalence [35]

Among middle-aged subjects (35–65 years old), sex ratios (M/F) for the prevalence of hypertension were generally lower than one (Table 1). However in three studies conducted in Lebanon [29], Palestine [32] and KSA [26], the reverse was true, with hypertension higher in males than females, and the difference statistically significant in the first two studies (*p* <0.001 and *p* = 0.027, respectively) [29, 32]. Patterns of age-gender disparities in hypertension varied across countries. In one group of studies (one conducted in Egypt, two in Jordan and three in Tunisia), hypertension was higher in females than males in the total sample size and at midlife [23–25, 34, 35, 37]. In other countries of the region (Algeria, KSA, Kuwait, Oman, and the UAE), overall prevalence rates of hypertension were higher among males, but prevalence was higher among women at midlife [21, 27, 28, 31, 39] with the three exceptions noted above [26, 29, 32].

Similar results were observed in three studies that provided only graphic representations of HBP prevalence among women and men: prevalence rates at midlife were higher among women than men in Algeria, Syria and Tunisia [22, 33, 38]. In Algeria, the sex ratio (M/F) was lower than one across all age categories including midlife [22]. In Syria [33], while M/F ratio was higher than one before midlife, the reverse was observed later on. In Tunisia, HBP was almost 1.5 times higher among females than males from 35 to 64 years of age [38].

**Fig. 2:**
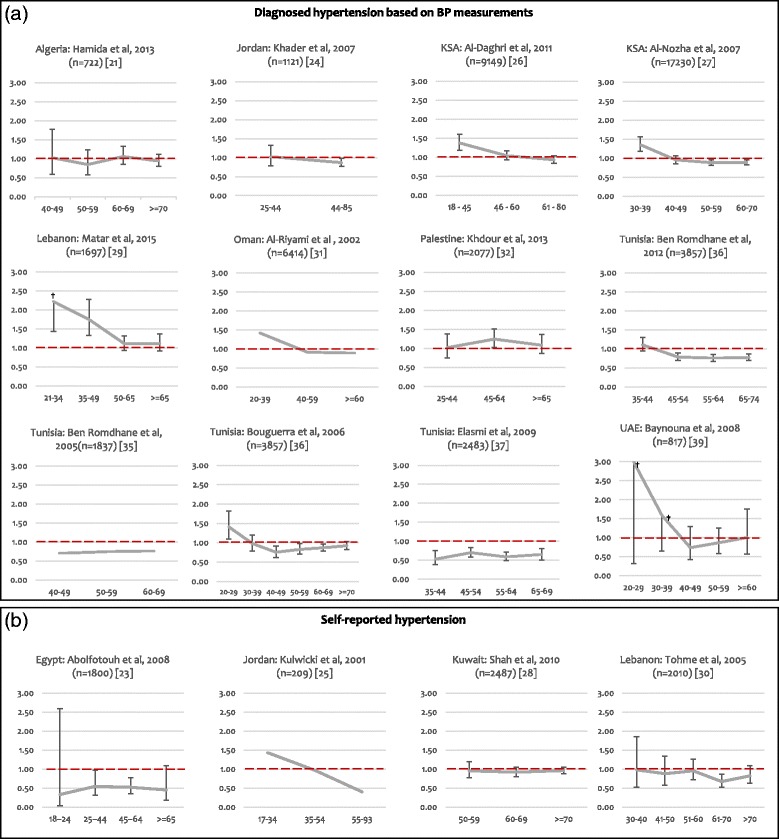
Sex Ratios (M/F) for the prevalence of hypertension by age category in the Arab world. Legend: BP: Blood pressure; F: Female; M: Male. **a** Hypertension was assessed by actual BP measurements [26, 27, 39], or by either BP measurements or self-report of previous diagnosis [21, 24, 29, 31, 32, 34–37]; **b** Hypertension was self-reported. The solid line corresponds to the M/F ratio by age group for each study. The dotted horizontal line corresponds to a M/F ratio of 1; solid lines above represent higher prevalence among males and solid lines below represent higher prevalence among females. The sample size for each study is shown in parenthesis. The error bars presented in the graphs correspond to the 95% CI of the sex ratios. The 95% CI in three studies [25], [31], and [35], could not be calculated due to insufficient data. ^†^ The upper limits of the 95% CI of the sex ratios were: 3.39 for the age group of 21–34 in Matar et al., 2015 [29], 28.01 and 4.00 for the age groups of 20–29 and 30–39, respectively, in Baynouna et al., 2008 [39].These values are not shown on the graphs, as we fixed the upper limit of the y-axis to 3, in order to keep the same scale for all graphs and allow comparisons

Figure 2 provides a graphic illustration of gender disparities and shows M/F ratios for the prevalence of hypertension by age group. The dotted horizontal line corresponds to a M/F ratio of one; solid lines above represent higher prevalence among males and solid lines below represent higher prevalence among females. Most studies showed ratios <1 for years corresponding to midlife. In studies that included subjects younger than 35 years old [23–27, 29–32, 36, 39], M/F ratios decreased at midlife, except in Palestine [32], where the sex ratio increased during midlife, with a significant difference between genders (p = 0.027). In Jordan [24, 25], KSA [27], Oman [31], Tunisia [36] and the UAE [39], M/F ratios were initially higher than one at younger ages, but this pattern was reversed at midlife when M/F ratios decreased to less than one. On the other hand, in one study conducted in Lebanon [29], the M/F ratio decreased during midlife but remained >1; and in one study conducted in KSA [26], the sex ratio decreased, remaining >1 among middle-aged subjects, and decreasing to <1 among the elderly.

## Discussion

This is the first review to analyze gender disparities in hypertension among middle-aged Arabs. We were able to retrieve 19 articles reporting on the prevalence of hypertension in the Arab region, by gender and age category, including the age range of 35 to 65 years. The paucity of research is remarkable, given that hypertension is the leading risk factor for morbidity and mortality in the region [3, 19].

A large body of evidence shows that gender disparities in HBP are age-dependent, with women witnessing a steeper increase in hypertension during midlife, as compared to men [7–11]. Cross-sectional studies such as the NHANES 2007–2012 [40, 41], the Community Hypertension Evaluation Clinic Program [42], and the Hypertension Detection and Follow-up Program Cooperative Group [43] indicate that blood pressure rises more steeply among middle-aged women than men, and that HBP rates in women surpass those of men around the fifth and sixth decades of life [15]. The Framingham Heart study also showed that blood pressure rates increased in middle-aged women, although they did not exceed those of men at any age between 30 and 60 years of age [44].

In this review, and consistent with the evidence in most settings, we found that sex ratios (M/F) in the prevalence of HBP decreased at midlife in most Arab countries, indicating that blood pressure increases at midlife among Arab women. We also found that the overall prevalence of hypertension was higher among middle-aged women as compared to men. Importantly, the prevalence of HBP among women surpassed that of men starting in the 4^th^ decade of life, which is consistent with previous studies conducted in the Middle East [7], but different from studies conducted in the United States [12], Europe [45, 46], and China [47, 48], where rates of hypertension in women exceeded those of men only starting the 6^th^ decade of life.

The only Arab population where the M/F ratio increases during midlife is in Palestine. This finding is puzzling, not only because it is an exception compared to the rest of the countries of the region, but also because other evidence indicates that overweight and obesity, which are associated with higher blood pressure, are higher among middle-aged Palestinian women than men [49]. One can conjecture that in the context of continuing political and military conflict, men may be more exposed to stress and economic hardship than women, and hence more vulnerable to blood pressure increases. This would be in line with literature showing that stress is associated with increased blood pressure, probably via mechanisms involving excessive sympathetic nervous system activation and transient endothelial dysfunction which can lead to vasoconstriction [50].

The higher prevalence of HBP among women has been attributed to a number of biological and environmental factors [26]. Estrogen has been shown to have vasodilation properties and progesterone to induce vascular relaxation through various mechanisms [51–56]. In line with this evidence, menopause is frequently pinpointed as a risk factor for HBP in midlife women, since the protective effects of ovarian hormones on blood pressure gradually disappear with the onset of menopause. Several cross-sectional studies have reported significantly higher blood pressure in postmenopausal than premenopausal women [57, 58]. Nevertheless, the association of menopause with HBP is complex due to confounding factors such as age and body weight [9, 10, 15–18]. Evidence from few prospective studies [59–61] suggests that menopause per se is not associated with increases in BP. In the Arab world, menopause was significantly and positively associated with HBP in several cross-sectionals studies conducted in Lebanon [62], Bahrain [63], and North African countries [64–66].

Obesity is a major cause of elevated blood pressure [6] and is remarkably higher among Arab women than men at midlife [49]. Obesity and overweight are associated with higher risk of hypertension and mortality among women than men of the same age [67]. The mechanism behind this association is still a matter of debate, and research has considered a variety of biological and hormonal factors [6, 68]. Lack of physical activity can also contribute to HBP, and global comparisons show that physical inactivity in the Arab world is particularly high [69, 70]. A closer examination of possible differences in environmental or behavioral factors among Arab middle-aged women and men, such as exposure to stress or salt consumption, could provide further insights into the reasons behind these gender differences.

The fact that the reversal in the sex ratios occurs earlier in the Arab world than elsewhere (the 4^th^, compared to the 6^th^ decade of life) deserves further investigation. Some studies have documented that mean age at menopause is earlier in Arab countries than in countries of the North [71–73]. Earlier menopause may be a contributing factor, but is unlikely to fully account for this earlier onset of hypertension. In addition the prevalence rates of obesity, diabetes, and physical inactivity in women from the Middle East and North Africa (MENA) region are among the highest worldwide [49, 69, 74–76]. Other studies have shown that obesity prevalence rates are higher among women than men globally, and that the lowest M/F sex ratios are observed in the MENA region including at midlife [49, 77]. Clustering of elevated blood pressure, abdominal obesity, type 2 diabetes, and dyslipidemia has been well documented in the literature [78], and involves several complex mechanisms including insulin resistance, inflammation, oxidative stress, endothelial dysfunction, activation of the sympathetic nervous system and the renin-angiotensin-aldosterone system [79]. It may be that the higher rates of obesity, diabetes, and physical inactivity observed among Arab Women as compared to global figures, contribute to explaining the earlier onset of hypertension and reversal of sex ratios observed in the Arab region. Further investigations that consider hypertension, together with other genetic and social determinants would throw light on other possible reasons.

The findings of this review should be considered in light of the following limitations. First, the review is based on a relatively small number of studies, and although the results appear to be consistent across studies, they cannot be taken to be representative of the region. Secondly, the prevalence of hypertension was not available for the same age categories across all the studies, hence comparative statistics have to be taken with caution. Thirdly, the statistical significance of gender differences was calculated based on information provided in the articles and we did not have access to the data themselves. Fourthly, it is possible that in those four studies where hypertension was based on self-reports, the higher prevalence among women could be attributed to women’s tendency to self-report their medical conditions to a greater extent than men [8]; this would not, however, explain why the same gender differences are also found in studies that relied on measured hypertension. Fifth, studies that met the inclusion criteria were all cross-sectional, and thus comparing the prevalence of hypertension in men and women across the life span is likely to be confounded by the changes in representation of the surviving population. This highlights the need for longitudinal studies that would allow comparing the same study population over time [15].

## Conclusions

This review has analyzed the available evidence on hypertension by gender during midlife, among Arab populations. The relatively small number of articles that met inclusion criteria highlights an important research gap, particularly in view of the risk factor that HBP represents in the Arab world. M/F ratios decrease at midlife in most Arab countries, and underscore the importance of refuting the misconception that hypertension is a “man’s disease.” The increase in prevalence of hypertension among women occurs earlier than in other regions; this pattern deserves further investigation. Studies that consider biological factors, behaviors, and the socio-cultural context of the Arab region are needed in order to understand the reasons behind such gender differences and identify those factors that could be modified in order to formulate programs designed to improve well-being among women and men.

## Abbreviations

DALYs: Disability-adjusted life years
DBP: Diastolic blood pressure
F: Female
HBP: High blood pressure
KSA: Kingdom of Saudi Arabia
M: Male
MENA: Middle East and North Africa
NHANES: National Health and Nutrition Examination Survey
SBP: Systolic blood pressure
SSCI: Social Sciences Citation Index
UAE: United Arab Emirates
